# Cyclic-di-GMP Regulates Autoaggregation Through the Putative Peptidoglycan Hydrolase, EagA, and Regulates Transcription of the *znuABC* Zinc Uptake Gene Cluster in *Erwinia amylovora*

**DOI:** 10.3389/fmicb.2020.605265

**Published:** 2020-11-17

**Authors:** Roshni R. Kharadi, George W. Sundin

**Affiliations:** Department of Plant, Soil and Microbial Sciences, Michigan State University, East Lansing, MI, United States

**Keywords:** fire blight, *Erwinia amylovora*, zinc, Zur, ZnuABC, autoaggregation, peptidoglycan hydrolase

## Abstract

*Erwinia amylovora* is the causal agent of fire blight, an economically impactful disease that affects apple and pear production worldwide. *E. amylovora* pathogenesis is comprised of distinct type III secretion-dependent and biofilm-dependent stages. Alterations in the intracellular levels of cyclic-di-GMP (c-di-GMP) regulate the transition between the different stages of infection in *E. amylovora*. We previously reported that hyper-elevation of c-di-GMP levels in *E. amylovora* Ea1189, resulting from the deletion of all three c-di-GMP specific phosphodiesterase genes (Ea1189Δ*pdeABC*), resulted in an autoaggregation phenotype. The two major exopolysaccharides, amylovoran and cellulose, were also shown to partially contribute to autoaggregation. In this study, we aimed to identify the c-di-GMP dependent factor(s) that contributes to autoaggregation. We conducted a transposon mutant screen in Ea1189Δ*pdeABC* and selected for loss of autoaggregation. Our search identified a peptidoglycan hydrolase, specifically, a D, D-endopeptidase of the metallopeptidase class, EagA (*Erwinia* aggregation factor A), that was found to physiologically contribute to autoaggregation in a c-di-GMP dependent manner. The production of amylovoran was also positively affected by EagA levels. An *eagA* deletion mutant (Ea1189Δ*eagA*) was significantly reduced in virulence compared to the wild type *E. amylovora* Ea1189. *eagA* is part of the *znuABC* zinc uptake gene cluster and is located within an operon downstream of *znuA.* The *znuAeagA*/*znuCB* gene cluster was transcriptionally regulated by elevated levels of c-di-GMP as well as by the zinc-dependent transcriptional repressor Zur. We also observed that with an influx of Zn^2+^ in the environment, the transcription of the *znuAeagA/znuBC* gene cluster is regulated by both Zur and a yet to be characterized c-di-GMP dependent pathway.

## Introduction

Fire blight is a bacterial disease that affects apple production with severe economic implications in the major apple growing regions around the world (Smits et al., 2017). *Erwinia amylovora* is the pathogen responsible for causing fire blight, and uses a wide range of virulence factors to infect its host in several distinct stages (Smits et al., 2017). Shoot infection is initiated by the entry of *E. amylovora* cells through microscopic wounds or natural openings in leaves at shoot tips. Once in the apoplast, *E. amylovora* uses type III secretion to implement effector-mediated virulence, which is outwardly manifested in the form of necrotic lesions on the plant surface (Zhao et al., 2009; Smits et al., 2017). Following this, *E. amylovora* invades the xylem, where it can attach to the walls of xylem vessels and proliferate rapidly and extensively to form robust biofilms, that block water passage though the xylem channels (Koczan et al., 2009, 2011; Castiblanco and Sundin, 2016) resulting in wilting and shoot blight symptoms in the host.

Cyclic-di-GMP (c-di-GMP) is a ubiquitous bacterial second messenger molecule (Romling et al., 2013) that signals the transition between the type III secretion and biofilm phases of infection in *E. amylovora* (Edmunds et al., 2013; Kharadi et al., 2019). Diguanylate cyclase enzymes (Dgcs) can sense a wide range of environmental signals and enzymatically regulate the synthesis of c-di-GMP intracellularly, through an active GGDEF domain (Romling et al., 2013). In contrast, phosphodiesterase enzymes (Pdes) with an active EAL domain, regulate the hydrolysis of c-di-GMP into the molecule 5′-phosphoguanylyl-(3′ → 5′)—guanosine (pGpG) (Romling et al., 2013). Varying intracellular levels of c-di-GMP impact key virulence factors in *E. amylovora*, including, but not limited to the type III secretion system (Edmunds et al., 2013; Kharadi et al., 2019). Biofilm formation, and the production of the major biofilm exopolysaccharides amylovoran and cellulose are also positively regulated through c-di-GMP signaling in *E. amylovora* (Edmunds et al., 2013; Romling et al., 2013; Castiblanco and Sundin, 2018; Kharadi et al., 2019).

Elevated intracellular levels of c-di-GMP have been implicated in inducing aggregative cellular behavior in both gram-positive and gram-negative bacteria. For example, c-di-GMP was found to regulate the transcription of the type IV pilus-related genes *pilA1* and *pilB1* in *Clostridium difficile* via a riboswitch, with cell aggregation being the downstream effect of the elevated transcription of these genes (Purcell et al., 2012; Bordeleau et al., 2015). Also, elevated intracellular levels of c-di-GMP were linked to an autoaggregative physiological condition in *Burkholderia pseudomallei*, *Pseudomonas aeruginosa*, and *Thermosynechococcus elongatus* (Borlee et al., 2010; Lee et al., 2010; Enomoto et al., 2014). In our previous study, we found that the deletion of the three active phosphodiesterase enzyme encoding genes, *pdeA*, *pdeB*, and *pdeC* in *E. amylovora* Ea1189, resulted in an approximately 10-fold increase in intracellular c-di-GMP levels (Kharadi et al., 2019). While amylovoran production was elevated in the Ea1189Δ*pdeABC* strain and flagellar motility was significantly reduced, biofilm formation under static conditions was reduced relative to other single and double *pde* deletion mutants with comparatively lower intracellular levels of c-di-GMP (Kharadi et al., 2019). This disconnect was attributed to the severe autoaggregation phenotype elicited in the Ea1189Δ*pdeABC* strain, which negatively impacted the ability of the cells to interact with a surface due to elevated cell-cell interactions (Kharadi and Sundin, 2019).

We also observed that several cells of Ea1189Δ*pdeABC* within an aggregate cluster were elongated and filamentous due to an impairment in cell separation post cell division and correlated this phenotype with an overall reduction in *ftsZ* transcript levels (Kharadi and Sundin, 2019). The activity of peptidoglycan hydrolases has been linked to the ability of daughter cells to properly separate post division in *Listeria monocytogenes, Clostridium perfringens* and *Streptococcus pneumoniae* (Carroll et al., 2003; Camiade et al., 2010; Sham et al., 2011). The endopeptidase class of peptidoglycan hydrolases cleaves amide bonds between amino acids within peptidoglycan to enable cell wall remodeling during growth (Vollmer et al., 2008). Once such endopeptidase is MepM (formerly YebA) in *Escherichia coli*, a metal dependent endopeptidase (Graham et al., 2009; Singh et al., 2012). MepM homologs include LytM (Singh et al., 2010), lysostaphin (Wu et al., 2003), and ALE-1 (Sugai et al., 1997) in *Staphylococcus aureus*, and these enzymes function through the cleavage of pentaglycine interpeptide bridges within peptidoglycan chains. Another MepM homolog, ShyB, is a zinc-dependent endopeptidase in *Vibrio cholerae* that is selectively expressed during conditions of zinc starvation and is comprised of a LysM domain and a M23 metallo-endopeptidase domain. *shyB* is under the transcriptional regulation of Zur, a regulator that belongs the Fur-regulator family, and is thought to function as an alternative endopeptidase to enable growth under zinc starvation conditions (Patzer and Hantke, 2000; Hantke, 2002; Murphy et al., 2019).

Zur is a repressor that functions as part of the overall zinc starvation response in gram positive and gram-negative bacteria (Patzer and Hantke, 2000; Shin et al., 2007). Zur contains two Zn^2+^ binding sites that are required for its overall stability and for its DNA-binding ability at Zur boxes present in the promoter regions of Zur regulon genes (Gilston et al., 2014). A well-studied target of Zur-mediated regulation is that of the ZnuABC zinc uptake system. Zinc is an essential element for bacterial survival, and an integral component of several enzymes and proteins (Hantke, 2005). ZnuABC and ZinT are two major bacterial zinc uptake regulation factors (Gabbianelli et al., 2011). Within the *znuABC* gene cluster, ZnuA is a periplasmic zinc binding protein, while ZnuB is the integral membrane import channel protein, and is associated with the ATPase ZnuC. *znuA* and *znuCB* are located adjacent to each other, but are transcribed in opposite directions (Patzer and Hantke, 2000; Gilston et al., 2014). The short intergenic region separating *znuA* and *znuC* contains σ^70^-regulated promoter sequences and encompasses the Zur binding box (Patzer and Hantke, 2000; Gilston et al., 2014; Mikhaylina et al., 2018). Additionally, in *E. coli*, the peptidoglycan hydrolase encoding gene, *mepM*, is located in an operon and is co-transcribed with *znuA* (Graham et al., 2009). In contrast, *shyB* in *V. cholerae* is transcribed as a separate gene but is also transcriptionally regulated by Zur (Murphy et al., 2019). These metallopeptidases have been linked to the *znuABC* gene cluster, and their function is dependent upon their interactions with Zn^2+^ cations (Graham et al., 2009; Murphy et al., 2019). The ZnuABC zinc uptake system is critical for virulence in *Salmonella enterica* (Campoy et al., 2002; Ammendola et al., 2007), *E. coli* (Gabbianelli et al., 2011), and *Yersinia pestis* (Bobrov et al., 2014; Bobrov et al., 2017), especially during conditions of metal starvation, a host defense strategy, whereby the freely available reserves of critical metals such as iron and zinc are severely limited (Schaible and Kaufmann, 2005). In *E. coli*, Zur functions to repress expression of *znuA* and *znuCB* when Zn^2+^ is available (Patzer and Hantke, 2000; Gilston et al., 2014; Mikhaylina et al., 2018).

In our previous study, we found that autoaggregation was dependent on intracellular c-di-GMP levels in *E. amylovora*, and that the exopolysaccharides amylovoran and cellulose partially contributed to autoaggregation (Kharadi and Sundin, 2019). Leading into this current study, we hypothesized that a cyclic-di-GMP dependent factor was involved in the regulation of autoaggregation in *E. amylovora*. We conducted a transposon screen to select for a loss of autoaggregation in *E. amylovora* under high intracellular levels of c-di-GMP. Our results identified that a MepM/ShyB homolog EagA, a peptidoglycan hydrolase of the DD-endopeptidase class, was required for autoaggregation. We found that EagA contributes to amylovoran production and virulence in *E. amylovora*. We also found that *eagA* was located adjacent to and co-transcribed with *znuA*, and that the *znuAeagA* as well as *znuCB* operons were transcriptionally regulated by Zur as well as by c-di-GMP.

## Materials and Methods

### Bacterial Strains, Plasmids, and Growth Conditions

All of the relevant bacterial strains and plasmids involved in the study are listed in Table 1. *E. amylovora* and *Escherichia coli* strains were grown in Luria-Bertani (LB) medium at 28 and 37°C respectively. For amylovoran quantification and *amsG* transcript level measurement using q-RT-PCR, strains were grown in MBMA medium (minimal medium amended with 1% sorbitol) (Edmunds et al., 2013). Cells were resuspended in SG (10 g sorbitol, 2 g L-glutamic acid, 0.5 g KH_2_PO_4_, 0.2 g NaCl, 0.2 g MgSO_4_.7H_2_O per liter, pH 7.0) medium amended with 200 nM ZnSO_4_ or 200 nM N,N,N′,N′-tetrakis (2-pyridinylmethyl)-1,2-ethanediamine (TPEN) (McCabe et al., 1993) to measure the transcript levels of the *znuABC*/*eagA* gene cluster, as well as to determine the autoaggregation factor. The media were also amended as appropriate with antibiotics: ampicillin (Ap; 100 μg/ml), chloramphenicol (Cm; 10 μg/ml), gentamicin (Gm; 10 μg/ml), kanamycin (Km; 100 μg/ml), or tetracycline (Tc; 10 μg/ml), and with 1 mM isopropyl β-D-1-thiogalactopyranoside (IPTG) to induce any relevant gene overexpression vectors.

**TABLE 1:** Bacterial strains and plasmids used in this study and their relevant characteristics.

Bacterial strain or plasmid	Relevant characteristics	Source or references
*E. amylovora* strains		
Ea1189	Wild type	Edmunds et al., 2013
Ea1189Δ*pdeABC*	*pdeA*, *pdeB* and *pdeC* deletion mutant	Kharadi et al., 2019
Ea1189ΔeagA	*eagA* deletion mutant	This study
Ea1189Δ*pdeABC*Δ*eagA*	*pdeA*, *pdeB*, *pdeC* and *eagA* deletion mutant	This study
Ea1189Δ*zur*	*zur (EAM_0267)* deletion mutant	This study
Ea1189Δ*pdeABC*Δzur	*pdeA*, *pdeB*, *pdeC* and *zur* deletion mutant	This study
*E. coli* strains		
S17-1	Strain carrying Tn*5* B30 Tet^*R*^	Simon et al., 1989
Plasmids		
pKD3	Cm^*R*^ cassette flanking FRT sites; Cm^R^*	Datsenko and Wanner, 2000
pKD46	L-Arabinose-inducible lambda red recombinase; Ap^*R*^	Datsenko and Wanner, 2000
P TL18	IPTG-inducible FLP; Tet^R^**	Long et al., 2009
pBBR1MCS-5	Broad-host-range cloning vector; R6K ori; Gm^*R*^	Kovach et al., 1995
pEVS143	Broad-host-range, IPTG inducible (Ptac) cloning vector; inducible Cm^*R*^ and GFP Km^*R*^	Dunn et al., 2006
pACYCDuet-1	Expression vector containing two MCS: P15A ori; Cm^R^***	Novagen (Darmstadt, Germany)
pRRK02 (In text: *ppdeB*)	*pdeB* with native promoter in pBBR1MCS-5; Gm^*R*^	Kharadi et al., 2019
pRRK06 (In text: *ppdeAC*)	*pdeA* and *pdeC* with their respective native promoters in pACYCDuet-1; Cm^*R*^	Kharadi et al., 2019
pRRK10 (In text: *peagA*)	*eagA* with native promoter in pBBR1MCS-5; Gm^*R*^	This study
pRRK11 (In text: *peagA* OE)	*eagA* in pEVS143; Km^*R*^	This study
pRRK12 (In text: *pzur*)	*zur* with native promoter in pBBR1MCS-5; Gm^*R*^	This study
pVC_DGCOE	*V. cholerae* gene VCA0956 in pEVS143; Km^*R*^	Waters et al., 2008

*IPTG, isopropyl-β-D-thiogalactopyranoside; **FRT, flippase target recognition; ***MCS, multiple cloning site.

### Bioinformatics

All aligned genome sequence and annotation files for *E. amylovora* ATCC 49946 were acquired from National Center for Biotechnology Information (NCBI) (Sebaihia et al., 2010). Artemis (Java) was used to browse through the genome and gather data about any gene accession IDs, open reading frame (ORF) organization, and individual gene nucleotide and protein sequences for the purposes of genetic manipulation and analysis. Pfam version 32.0 was used for protein domain annotation and analysis (El-Gebali et al., 2019). MEGA version 7.0 was used for protein/DNA alignments (Kumar et al., 2016).

### Genetic Manipulation and Analysis

DNA manipulations were conducted using standard protocols (Sambrook and Russell, 2001). Chromosomal deletion mutants in *E. amylovora* were constructed using the lambda red recombinase system-based protocol as previously described (Datsenko and Wanner, 2000). *zur*, *pdeB*, and *eagA* were cloned into the broad host range, low copy number vector pBBR1-MCS5 (Kovach et al., 1995) along with their native promoter region for the complementation of Ea1189Δ*pdeABC* or Ea1189Δ*eagA* strains (single or combination mutant strains). Both *pdeA* and *pdeC* were cloned along with their native promoter region into pACYC-Duet1 vector into each of the two MCS within the vector. The ORF region of *eagA* was cloned into pEVS143 for inducible expression using IPTG. All relevant oligonucleotide primers used in the study are listed in Supplementary Table 1.

### Transposon Mutagenesis and Mutant Analysis

Transposon mutagenesis was conducted through bi-parental mating between Ea1189Δ*pdeABC* and *Escherichia coli* S17-1 carrying Tn*5-*B30 as previously described (Simon et al., 1989; Erickson et al., 2016). Transposon mutant libraries were plated onto LB agar-based medium amended with ampicillin and tetracycline, and individual colonies were inoculated into single wells within 96-well plates containing LB liquid medium (amended with the aforementioned antibiotics), and grown at 28°C for 48 h with gentle shaking. Mutants with a visual total loss of autoaggregation were selected and confirmed to have lost the autoaggregation phenotype after overnight growth in LB broth. The identification of transposon insertion sites in the selected group of mutants was conducted using an arbitrary PCR based approach as previously described (Lauro et al., 2008). Arbitrary PCR was used to expand the Tn flanking regions using the primers described in Supplementary Table 1, and Sanger sequencing was done through both flanking ends. The sequences were then compared to the *E. amylovora* ATCC-49946 genome via BLAST to locate the Tn insertion site on the chromosome (Sebaihia et al., 2010).

### Visual Analysis of Autoaggregation Using Scanning Electron Microscopy

To analyze the cell growth patterns and autoaggregate physiology, strains were grown in LB liquid medium amended with the appropriate antibiotics and IPTG as required for 18 h at 28°C. Following incubation, cell samples were collected from the culture tubes and fixed with 2.5% paraformaldehyde-2.5% glutaraldehyde. Following this, samples were dehydrated at progressively increasing ethanol concentrations, critical point dried, and osmium coated as previously described (Kharadi and Sundin, 2019). The JEOL-JSM-7500F (cold field emission emitter) scanning electron microscope (Japan Electron Optics Laboratory Ltd., Tokyo, Japan) was used to visualize the samples.

### Quantification of Amylovoran Production and Aggregation Factor

Amylovoran production was quantified via a Cetylpyridinium Chloride (CPC) binding turbidimetric assay as previously described (Bellemann et al., 1994). Cells were grown in MBMA medium amended with sorbitol for 48 h at 28°C, following which CPC (50 mg/ml) was added to the supernatant. Finally, the ratio of OD_600_ of CPC based precipitation to the OD_600_ of the cell concentration was turbidometrically determined to quantify the relative levels of amylovoran production among the strains. To determine the aggregation factor, strains were grown in liquid LB medium for 18 h at 28°C with shaking. To check for conditional autoaggregation, strains were grown in LB at 28°C overnight and sub cultured in SG medium amended with 200 nm ZnSO_4_ or 200 nm TPEN, followed by an incubation at 28°C for 18 h with agitation. Following this, the ratio of the OD_600_ post homogenization/OD_600_ pre homogenization for the cultures was determined as previously described (Kharadi and Sundin, 2019). Three biological replicates were performed for each of the assays. Statistical analysis including Tukey’s honestly significant difference (HSD) was conducted using JMP statistical software^TM^.

### RNA Isolation and q-RT-PCR

To determine relative *eagA* transcript levels, strains were grown in LB for 18 h at 28°C with agitation. To quantify relative transcript levels of *amsG*, strains were grown overnight in LB at 28°C with shaking, followed by resuspension in MBMA medium for 6 h. For the quantitative determination of *eagA*, *znuA*, *znuB*, and *znuC* targets, strains were either grown in LB for 18 h at 28°C with shaking and processed or, following this were resuspended in SG medium amended with 200 nm ZnSO_4_ or 200 nm TPEN (McCabe et al., 1993) as appropriate for 15 min or 2 h. Following this, RNA was extracted from the cultures using the Direct-zol RNA Miniprep kit method (Zymo Research, Irvine, CA, United States), followed by cDNA synthesis using the RT Kit (Applied Biosystems, Foster City, CA, United States). Quantitative PCR experiments were conducted using the SYBR green PCR master mix (Applied Biosystems, Foster City, CA, United States). *recA* was used as an endogenous control. Relative fold change was calculated using the delta C_*T*_ method (Rao et al., 2013). Each experiment consisted of three biological replicates with three internal technical replicates each. Statistical analysis including Tukey’s honestly significant difference (HSD) or students *t*-test were conducted using JMP statistical software^TM^.

### Virulence Assay in Apple Shoots

Apple shoot infection assays were conducted as previously described (Kharadi et al., 2019). Strains were grown overnight at 28°C in LB with shaking and were normalized to an OD_600_ of 0.2 using phosphate buffer saline (PBS). Young leaves on central shoots of apple trees (*Malus x domestica* cv. Gala on M9 rootstock) were inoculated by cutting between the peripheral veins with surgical scissors dipped in inoculum. External tissue necrosis along the leaves, petiole and stem was used as an indicator of virulence levels at 8 dpi. Statistical analysis including Tukey’s honestly significant difference (HSD) was conducted using JMP statistical software^TM^.

### Quantification of Intracellular Levels of c-di-GMP

Intracellular levels were quantified as previously described (Kharadi et al., 2019) using ultra performance liquid chromatography coupled with tandem mass spectrometry (UPLC-MS-MS). Strains were grown in LB medium amended with the appropriate antibiotics for 18 h at 28°C with agitation. Pelleted cells were lysed (using 40% acetonitrile and 40% methanol) for 15 min at −20°C. A standard gradient was established using synthesized c-di-GMP (Axxora Life Sciences Inc., San Diego, CA) Samples were processed on a Quattro Premier XE instrument (Waters Corp.; Milford, MA). Statistical analysis including Tukey’s honestly significant difference (HSD) was conducted using JMP statistical software^TM^.

### Quantification of Biofilm Formation

Biofilm formation was quantified using a modified version of a previously described protocol (Kharadi et al., 2019). Strains were grown overnight in LB at 28°C with agitation. The OD_600_ of the liquid cultures was determined and normalized to a final effective OD_600_ of 0.05 following a dilution in 0.5× LB amended with antibiotics and IPTG as appropriate. A Polypropylene bead (dia. 7 mm) (Cospheric LLC) was suspended in this diluted broth, followed by incubation for 48 h at 28°C. A 0.1% crystal violet solution was used to stain the beads for 1 h, followed by elution with 200 μL of elution solution (40% methanol and 10% glacial acetic acid). The OD_595_ was measured of the eluted solution. Three biological replicates with three technical internal replicates were included in this study. Statistical analysis including Tukey’s honestly significant difference (HSD) was conducted using JMP statistical software^TM^.

### Quantification of Relative Flagellar Motility

A swimming motility assay, as previously described, was used to compare relative levels of flagellar motility in the strains (Edmunds et al., 2013). Strains were grown overnight in LB at 28°C, followed by a normalization of the OD_600_ of the cultures. A 10 μL sterile pipette tip was immersed in the cultures and stabbed onto a swimming motility plate (10 g Tryptone, 5 g NaCl and 0.3% agar/liter). The plates were incubated 28°C for 24 h. The area of the resulting cell spread on the plate was evaluated using Image J (Schneider et al., 2012). Three biological replicates with at least three technical replicates were included in this study. Statistical analysis including Tukey’s honestly significant difference (HSD) was conducted using JMP statistical software^TM^.

## Results

### EagA Regulates Autoaggregation in *E. amylovora* in a c-di-GMP Dependent Manner

From our transposon mutant screen, we evaluated 4048 independent Tn*5-*B30 insertion mutants in *E. amylovora* Ea1189Δ*pdeABC* (high intracellular c-di-GMP), and identified 17 mutants (0.42%) that displayed a loss of the autoaggregation phenotype. EAM_1999 was the major gene candidate identified in the screen with five insertional mutants, each with a unique Tn*5-*B30 insertion within the coding sequence (Table 2). Other genes identified in this screen included EAM_1299, EAM_0370, EAM_2666 and EAM_1028. Findings about the involvement of these other genes and their contribution to the overall growth physiology of *E. amylovora* will be reported separately. EAM_1999 (new locus tag EAM_RS09640), formerly annotated as *mepM* (for its annotated functional characterization of being a murein endopeptidase), was renamed *eagA* (*Erwinia* aggregation factor A). For further phenotypic validation in this study, we constructed a chromosomal deletion mutant of *eagA* in *E. amylovora* Ea1189 and in the Ea1189Δ*pdeABC* background.

**TABLE 2:** Locations of transposon insertion sites from the autoaggregation loss screen for conducted in Ea1189Δ*pdeABC*.

Locus tag	Gene name*	Tn insertion site**	Annotated function*
EAM_1999	*eagA*	2148174	Murein DD-endopeptidase MepM
EAM_1999	*eagA*	2148193	Murein DD-endopeptidase MepM
EAM_1999	*eagA*	2147193	Murein DD-endopeptidase MepM
EAM_1999	*eagA*	2147714	Murein DD-endopeptidase MepM
EAM_1999	*eagA*	2148494	Murein DD-endopeptidase MepM

*Based on annotation of the ATCC 49946 genome (GenBank accession no. GCA_000027215.1). **Nucleotide position based on the ATCC 49946 genome (GenBank accession no. GCA_000027215.1). The nucleotide range for eagA is 2147267-2148592.

**FIGURE 1:**
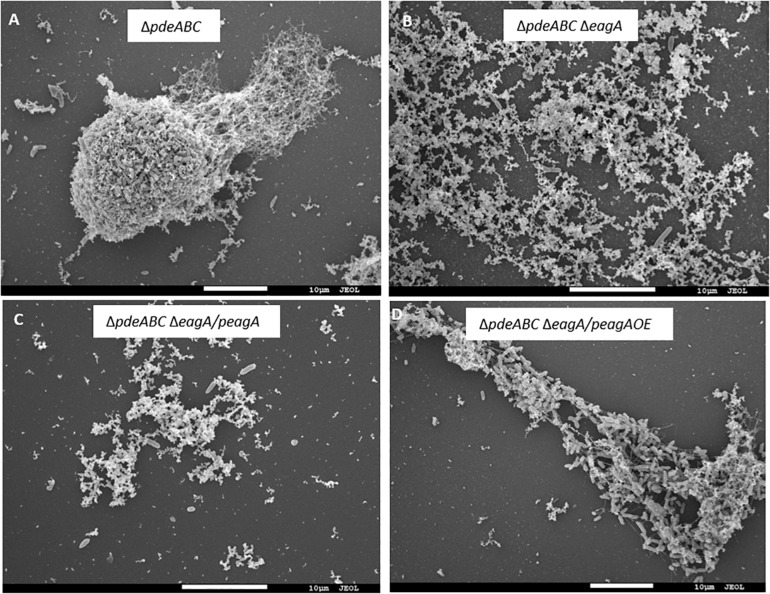
Scanning electron micrographs of *E. amylovora* Ea1189 deletion mutants following growth in LB for 18 h. **(A)** Ea1189Δ*pdeABC* displays elevated cell-cell interaction in the form of autoaggregation. **(B)** Ea1189Δ*pdeABC*Δ*eagA* cells show diffused organization, with homogenously interspersed EPS indicating a loss of autoaggregation. **(C)** Complementing Ea1189Δ*pdeABC*Δ*eagA* with *eagA* and its native promoter on a low copy number plasmid (p*eagA*) fails to fully restore autoaggregation. **(D)** Overexpression of *eagA* using 1 mM IPTG (p*eagA* OE) restores autoaggregation in Ea1189Δ*pdeABC*Δ*eagA*.

**FIGURE 2:**
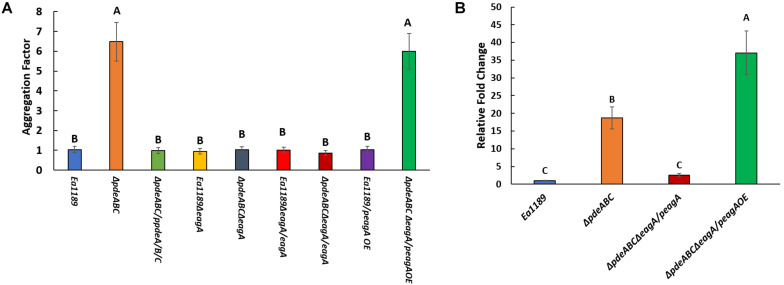
**(A)** Calculated aggregation factor for *E. amylovora* WT Ea1189, and Ea1189Δ*pdeABC*, Ea1189Δ*eagA*, and Ea1189Δ*pdeABC*Δ*eagA* mutant, complemented, and overexpression strains. Strains were grown LB with 1 mM IPTG as appropriate for 18 h prior to aggregation factor measurement. Data represent three biological replicates, and error bars represent standard error of the means. Differential letters above the bars indicate statistically significant differences [*P* < *0.05* by Tukey’s honestly significant difference (HSD) test]. **(B)** Relative fold change in *eagA* transcript levels measured by q-RT-PCR in WT Ea1189, Ea1189Δ*pdeABC*, complementation and overexpression strains. Strains were grown in LB for 18 h with 1 mM IPTG as appropriate prior to being processed. Data represent three biological replicates, and error bars represent standard error of the means. Differential letters above the bars indicate statistically significant differences [*P* < *0.05* by Tukey’s honestly significant difference (HSD) test].

After overnight growth in LB broth medium, *E. amylovora* Ea1189Δ*pdeABC* exhibited a severe autoaggregation phenotype, with a majority of the external EPS sequestered within the cell aggregate (Figure 1A). In contrast, Ea1189Δ*pdeABC*Δ*eagA* lost the autoaggregation phenotype, with cells showing an interspersed arrangement and no clear patterns of EPS clustering (Figure 1B). Complementation of Ea1189Δ*pdeABC*Δ*eagA* with *eagA* with its native promoter on a low copy number plasmid (p*eagA*) was not sufficient to fully complement the loss of autoaggregation (Figure 1C). Overexpression of *eagA* (p*eagA* OE; *eagA*:pEVS143 high copy number/tac promoter) with 1 mM IPTG for the duration of incubation resulted in the partial complementation of the autoaggregation phenotype in Ea1189Δ*pdeABC*Δ*eagA*, with cells displaying widespread clustering, with EPS binding to the clusters (Figure 1D). However, when *eagA* was overexpressed in WT Ea1189 (p*eagA* OE), autoaggregation was not observed (Figure 2A). Quantification of the aggregation factor of these strains *in vitro* yielded similar results. The calculated aggregation factor for Ea1189Δ*pdeABC* was significantly higher than WT Ea1189, while Ea1189Δ*pdeABC*Δ*eagA* displayed an aggregation factor similar to WT Ea1189 (Figure 2A). *eagA* transcript levels were significantly elevated in Ea1189Δ*pdeABC* compared to WT Ea1189, and, under the growth conditions that facilitate autoaggregation, the induced overexpression of *eagA* (through p*eagA*OE vector) was necessary to achieve comparable *eagA* transcript levels in Ea1189Δ*pdeABC*Δ*eagA* (Figure 2B). The deletion of *eagA* did not impact c-di-GMP levels in WT Ea1189 and Ea1189Δ*pdeABC* (Supplementary Figure 1).

### EagA Impacts Amylovoran Production in *E. amylovora*

**FIGURE 3:**
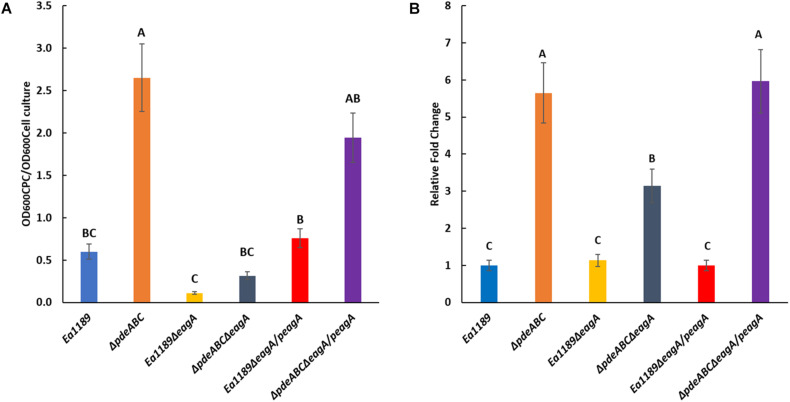
**(A)** Quantification of amylovoran production *in vitro* for WT *E. amylovora* Ea1189, and Ea1189Δ*pdeABC*, Ea1189Δ*eagA*, and Ea1189Δ*pdeABC*Δ*eagA* mutants and complemented strains after growth in MBMA medium for 48 h at 28°C. Data represent three biological replicates, and error bars represent standard error of the means. Differential letters above the bars indicate statistically significant differences [*P* < *0.05* by Tukey’s honestly significant difference (HSD) test]. **(B)** *amsG* transcript levels measured by q-RT-PCR *in vitro* for WT *E. amylovora* Ea1189, and Ea1189Δ*pdeABC*, Ea1189Δ*eagA*, and Ea1189Δ*pdeABC*Δ*eagA* mutants and complemented strains quantified after growth in MBMA medium for 6 h at 28°C. Data represent the fold change in transcript levels as compared to WT Ea1189, and include three biological replicates, and error bars represent standard error of the means. Differential letters above the bars indicate statistically significant differences [*P* < *0.05* by Tukey’s honestly significant difference (HSD) test].

The deletion of *eagA* resulted in a significant reduction of amylovoran production in Ea1189Δ*pdeABC*Δ*eagA* compared to Ea1189Δ*pdeABC*, as quantified through precipitation via cetylpyridinium chloride (CPC) binding (Figure 3A). Amylovoran production was also reduced in Ea1189Δ*eagA* compared to WT Ea1189, but the difference was not significant (Figure 3A). Complementation with *eagA* on pRRK10 recovered the amylovoran production levels in both Ea1189 and in Ea1189Δ*pdeABC.* We used the expression of *amsG* as a proxy for expression of amylovoran biosynthetic genes because *amsG* is the first gene of the amylovoran biosynthetic operon in *E. amylovora* (Koczan et al., 2009). *amsG* transcript levels were unaffected in Ea1189Δ*eagA* compared to WT Ea1189, but were significantly reduced in Ea1189Δ*pdeABC*Δ*eagA* compared to Ea1189Δ*pdeABC* (Figure 3B).

### EagA Positively Affects Virulence in *E. amylovora*

**FIGURE 4:**
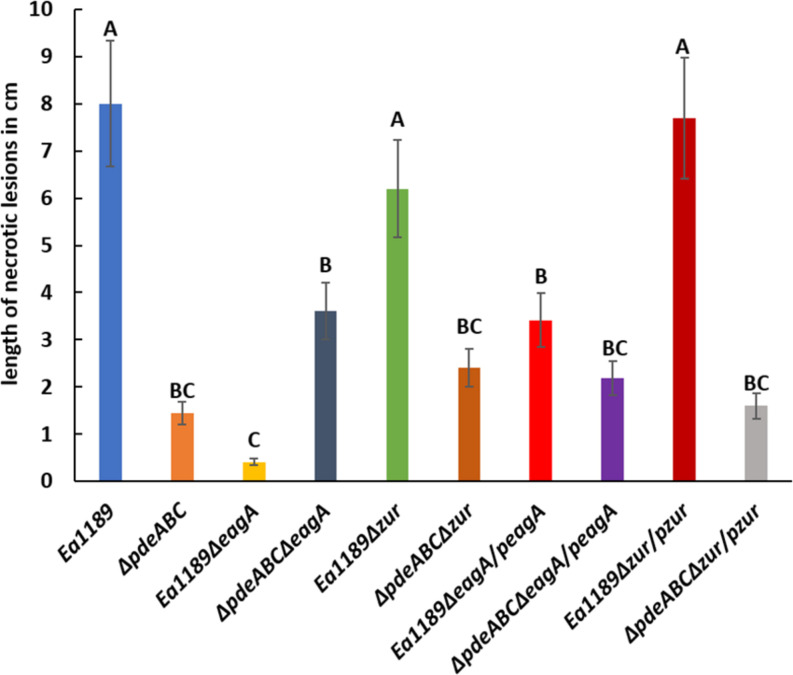
Quantitative analysis of virulence in apple shoots, measured in terms of necrotic lesion length along infected shoots, for WT *E. amylovora* Ea1189, and Ea1189Δ*pdeABC* and Ea1189Δ*eagA* mutants and complemented strains. Data represent three biological replicates, and error bars represent standard error of the means. Differential letters above the bars indicate statistically significant differences [*P* < *0.05* by Tukey’s honestly significant difference (HSD) test].

The deletion of *eagA* in Ea1189 resulted in a significant reduction in virulence compared to WT Ea1189 in an apple shoot infection assay (Figure 4). Complementation of Ea1189Δ*eagA* with *eagA* (pRRK10) partially restored virulence relative to WT Ea1189 (Figure 4). Limited by the dependence on IPTG induction for the overexpression of *eagA* (pRRK11), we were unable to test the effect of the overexpression of *eagA* in the apple shoot system. Although the length of necrotic lesions in apple shoots caused by Ea1189Δ*pdeABC*Δ*eagA* was significantly reduced compared to WT Ea1189, this mutant still caused significantly more disease than Ea1189Δ*eagA* (Figure 4). The deletion of *zur* did not yield any significant changes in virulence under the WT Ea1189 background or the Ea1189Δ*pdeABC* background (Figure 4).

### EagA Positively Regulates Biofilm Formation in a c-di-GMP Dependent Manner, and Negatively Regulates Flagellar Motility

**FIGURE 5:**
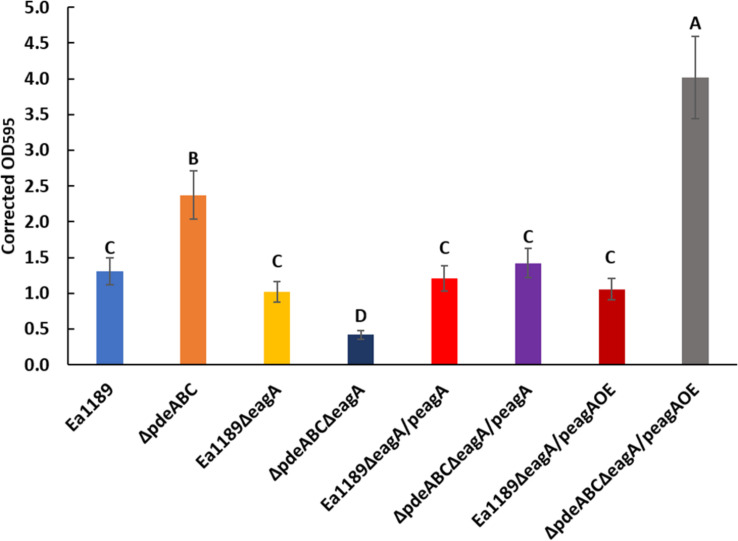
Relative levels of biofilm formation in WT *E. amylovora* Ea1189, Ea1189Δ*pdeABC* along with *eagA* mutants, complemented strains and overexpression strains in both backgrounds. Data presented in the form of comparative crystal violet binding levels includes three biological replicates with error bars representing the standard error of the means. Differential letters above the bars indicate statistically significant differences [*P* < *0.05* by Tukey’s honestly significant difference (HSD) test].

**FIGURE 6:**
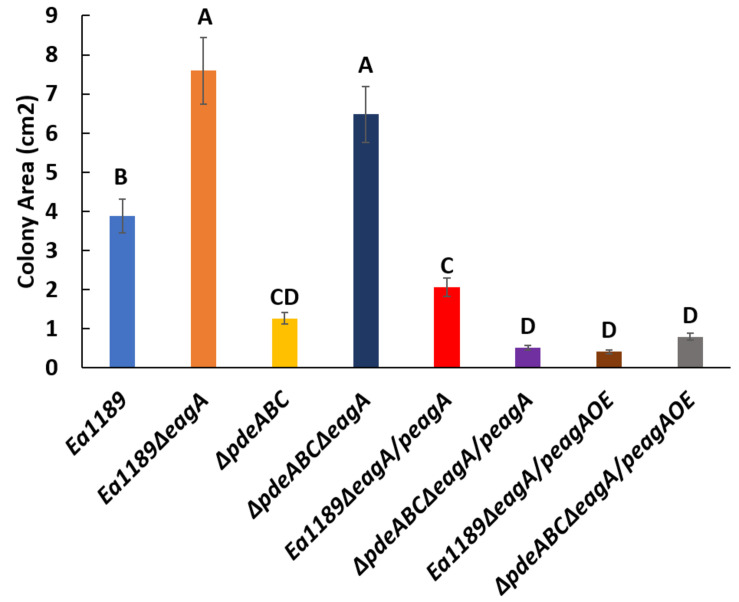
Relative levels of flagellar motility in WT *E. amylovora* Ea1189, Ea1189Δ*pdeABC* along with *eagA* mutants, complemented strains and overexpression strains in both backgrounds. The colony movement progression through low density motility agar plates is represented in the form of colony area (cm^2^). Data includes three biological replicates, and error bars represent standard error of the means. Differential letters above the bars indicate statistically significant differences [*P* < *0.05* by Tukey’s honestly significant difference (HSD) test].

The deletion of *eagA* in the Ea1189Δ*pdeABC* background resulted in a significant decrease in biofilm formation as compared to Ea1189Δ*pdeABC*. The decreased biofilm formation phenotype was partially complemented through the expression of the p*eagA* plasmid, and fully through the peagAOE vector (Figure 5). The deletion of *eagA* in WT Ea1189 did not yield any differences in biofilm formation (Figure 5). In addition, the deletion of *eagA* in both WT Ea1189 and Ea1189Δ*pdeABC* resulted in a significant increase in relative levels of flagellar motility (Figure 6). Complementation with the p*eagA* vector successfully enabled phenotypic restoration in the mutants (Figure 6).

### The *znuAeagA*/*znuCB* Gene Cluster Is Transcriptionally Regulated by Both Zur and c-di-GMP

**FIGURE 7:**
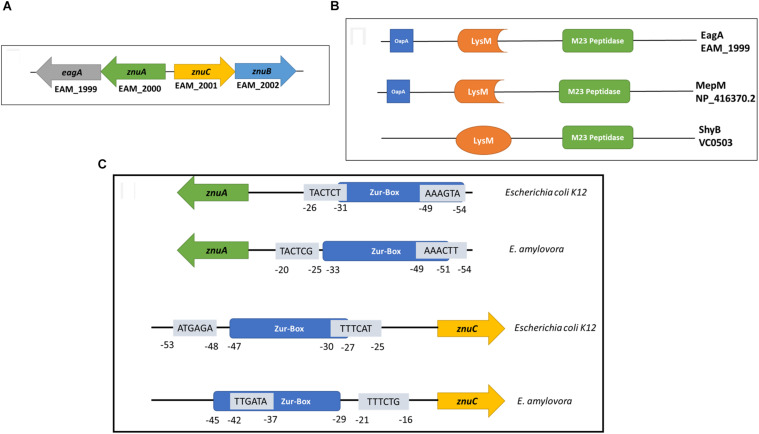
**(A)** Arrangement of the *znuABC* gene cluster and *eagA* in *E. amylovora* Ea1189. *znuA* and *eagA* are co-transcribed, and *znuCB* are co-transcribed. The intergenic region between *znuA* and *znuC* is 78 bp (Based on annotation of the ATCC 49946 genome) (Sebaihia et al., 2010) **(B)** EagA from *E. amylovora*, MepM from *E. coli* K12-W3110 (Hayashi et al., 2006) and ShyB from *V. cholerae* (Heidelberg et al., 2000) all contain a peptidoglycan binding LysM domain (Bateman and Bycroft, 2000) and a M23 metallo-endopeptidase domain (Grabowska et al., 2015). The percent amino acid identity between EagA and MepM is 69.5%, and the percent amino acid identity between EagA and ShyB is 35.2%. Protein domain annotation was acquired from Pfam version 32.0 (El-Gebali et al., 2019). MEGA version 7 was used for protein alignments (Kumar et al., 2016). **(C)** A representation of the bi-directional promoter contained within the *znuABC* gene cluster. Both *E. amylovora* and *E. coli* K12-W3110 have an intergenic region of 78 bp between *znuA* and *znuC*. Mapped and putative σ^70^ binding sites in both directions are presented in the figure with sequences in the 5′-3′ orientation, overlaid relative to the location of the consensus Zur-binding box (Hantke, 2002). The σ^70^ binding sites marked were previously mapped for *E. coli* K12 (Shimada et al., 2014) and have been correspondingly mapped for *E. amylovora*.

*EagA* is located in an operon with *znuA* in *E. amylovora* (Figure 7A). *znuC* and *znuB* (also co-transcribed) are located adjacent to *znuA* but are expressed in opposite orientation (Figure 7A). MepM in *E. coli* and ShyB in *V. cholerae* are homologs of EagA and share ∼70 and 35% amino acid identity with EagA, respectively (Figure 7B). In addition, all three proteins contain the peptidoglycan binding LysM domain and the M23 metallopeptidase domain (Figure 7B and Supplementary Figure 2). In *E. amylovora*, the intergenic region is 78 bp and contains a consensus Zur (transcriptional repressor) binding box at −55 to −33 relative to the translational start site of *znuA* (Figure 7C and Supplementary Figure 2). In both *E. amylovora* and *E. coli*, the bidirectional promoter contains σ^70^-consensus binding regions overlapping the Zur box. Due to the location of *eagA* as part of the *znuABC* gene cluster in *E. amylovora*, we decided to investigate the effect of c-di-GMP and Zur on the transcriptional regulation of *eagA* and *znuA* in the presence and absence of Zn^2+^. In our experiments, we used q-RT-PCR to measure the expression of *znuA*, *eagA*, and *znuC* in SG medium with two states relative to zinc: zinc depleted, due to the addition of 200 nm of TPEN or an adequate to excess zinc state due to the addition of 200 nm ZnSO_4_ at 15 min and 2 h of incubation.

**FIGURE 8:**
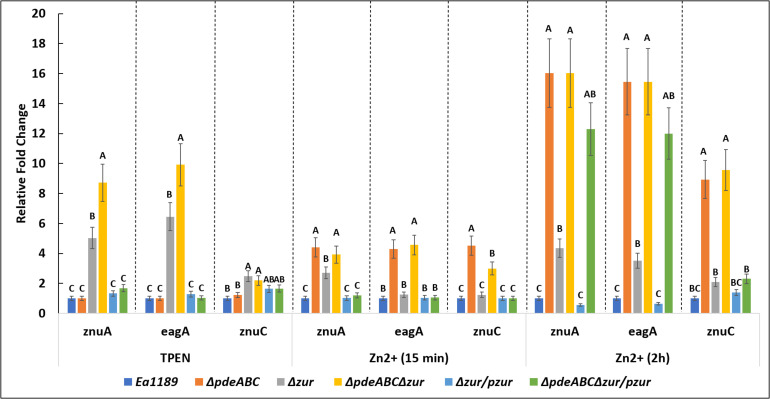
*eagA*, *znuA*, and *znuC* transcript levels measured by q-RT-PCR *in vitro* for WT *E. amylovora* Ea1189, and Ea1189Δ*pdeABC*, Ea1189Δ*zur*, and Ea1189Δ*pdeABC*Δ*zur* mutants and complemented strains after growth in SG medium amended with 200 nM TPEN or 200 nM ZnSO4 for15 min, and 2 h at 28°C. Data represent the fold change in transcript levels as compared to WT Ea1189 for each separate condition, and include three biological replicates; error bars represent standard error of the means. Differential letters above the bars indicate statistically significant differences [*P* < *0.05* by Tukey’s honestly significant difference (HSD) test] within each experimental condition subgroup.

In the absence of zinc (when chelated by TPEN), a loss of transcriptional repression of *znuA* and *eagA* was observed upon the deletion of *zur*, both under basal or elevated c-di-GMP containing strains (WT and Ea1189Δ*pdeABC*, respectively) (Figure 8). Ea1189Δ*pdeABC*Δ*zur* displayed further elevated transcript levels of *znuA* and *eagA* as compared to Ea1189Δ*zur.* Fifteen minutes after the influx of zinc, we observed a significant elevation in the transcript levels of *znuA*, *eagA* and *znuC* in the Ea1189Δ*pdeABC*Δ*zur* and Ea1189Δ*pdeABC* backgrounds compared to the other strains evaluated, including Ea1189Δ*zur* (Figure 8). In these strains, at this stage, as compared to WT Ea1189, the fold change for each of the three gene targets was within a 6-fold range of change on average. When we sustained the increase in zinc levels for 2 h, the level of sustained transcriptional elevation of *znuA*, *eagA* and *znuC* surpassed the 8-fold range in Ea1189Δ*pdeABC* and Ea1189Δ*pdeABC*Δ*zur*. Ea1189Δ*zur* also showed a higher rate of elevation of the three target genes, as compared to WT Ea1189 at 2 h post incubation (Figure 8). Additionally, the deletion of *zur* did not impact c-di-GMP levels under WT Ea1189 and Ea1189Δ*pdeABC* conditions (Supplementary Figure 1).

**FIGURE 9:**
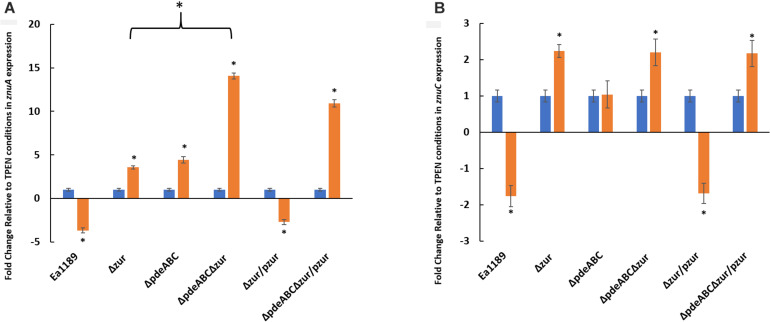
**(A)** *znuA* and **(B)** *znuC* transcript levels measured by q-RT-PCR *in vitro* for WT *E. amylovora* Ea1189, and Ea1189Δ*pdeABC*, Ea1189Δ*zur*, and Ea1189Δ*pdeABC*Δ*zur* mutants and complemented strains after growth in SG medium amended with 200 nM TPEN or 200 nM ZnSO4 for 2 h at 28°C. Data represent the fold change in transcript levels as compared to the TPEN condition for each separate strain for each of the target genes, and include three biological replicates; error bars represent standard error of the means. Asterisks above the bars indicate statistically significant differences in expression under the presence of Zinc vs. TPEN [*P* < *0.05* by student’s *t*-test] within each experimental subgroup.

In order to visualize the data contained in Figure 9 to be able to compare the effect of zinc addition and zinc chelation in the mutants and WT Ea1189, analysis of *znuA* and *znuC* expression was also done comparing expression in various mutants to that of the WT Ea1189 in both the zinc-depleted and zinc-adequate conditions. In zinc-depleted conditions, we observed a significant increase in *znuA* in Ea1189Δ*zur* and Ea1189Δ*pdeABC*Δ*zur*; complementation with *zur* reduced transcript levels to that of the WT Ea1189 in both cases (Figure 9A). In zinc-adequate conditions, Ea1189Δ*pdeABC* exhibited a significant, 16-fold increase in *znuA* expression compared to WT Ea1189, and the increase in expression was similar with or without the additional Δ*zur* deletion (Figure 9A). When compared to WT Ea1189, again the magnitude of increases in expression of *znuC* in Ea1189Δ*zur* and Ea1189Δ*pdeABC*Δ*zur* was lower than that for *znuA*, and *znuC* expression in Ea1189Δ*pdeABC*Δ*zur* was not significantly elevated compared to Ea1189Δ*zur* (Figure 9B). The expression pattern for *znuC* in Ea1189Δ*zur* and Ea1189Δ*pdeABC*Δ*zur* in the zinc-adequate condition was similar to that for *znuA* (Figure 9B). To determine if the transcriptional impact of elevated intracellular c-di-GMP levels was independent of both the inclusion of zinc as a potential signaling factor, and of its physiochemical specificity to a native *E. amylovora* pool of c-di-GMP, we examined the expression of *eagA*, *znuA*, *znuB*, and *znuC* upon the heterologous overexpression of the diguanylate cyclase encoding gene VCA0956 from *Vibrio cholerae* (pVC_DGCOE) in the WT Ea1189 background. Transcription of the evaluated gene targets did not show any significant differences in Ea1189/pVC_DGCOE strain when compared to WT Ea1189 (Supplementary Figure 3).

### Autoaggregation Requires the Presence of Zinc

**FIGURE 10:**
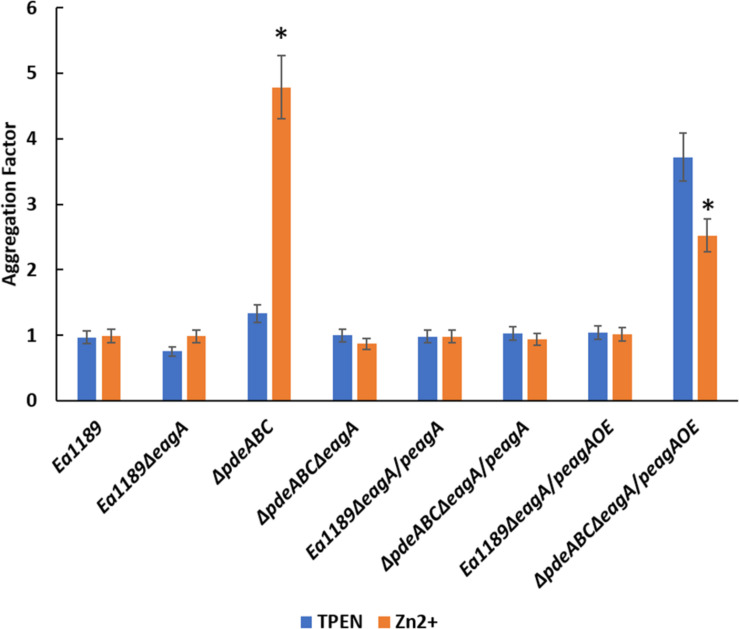
Aggregation factor for WT *E. amylovora* Ea1189, Ea1189Δ*pdeABC* along with *eagA* mutants, complemented strains and overexpression strains in both backgrounds, under zinc chelated (200 nm TPEN) or zinc abundant (200 nm ZnSO_4_) conditions. Strains were grown LB and sub-cultured for 18 h in SG medium amended with TPEN, ZnSO_4_, and IPTG as appropriate. Data includes three biological replicates, error bars representing the standard error of the means. Asterisk above the bars indicate statistically significant differences in aggregation under the presence of TPEN vs. zinc [*P* < *0.05* by student’s *t*-test] within each strain subgroup.

Having observed the negative effect of zinc chelation (with TPEN) on the transcriptional activity of *eagA* in Ea1189Δ*pdeABC* (Figures 8, 9), we evaluated the effect of zinc chelation on autoaggregation, by measuring the aggregation factor metric. The chelation (with 200 nm TPEN) or addition (with 200 nm ZnSO_4_) of zinc did not affect the aggregation factor under WT Ea1189 conditions, both in the presence and absence of *eagA* (Figure 10). In Ea1189Δ*pdeABC*, the chelation of zinc with TPEN significantly reduced the level of autoaggregation from that observed upon the addition of zinc (Figure 10). The reliance of the physiological manifestation of autoaggregation on the transcriptional abundance of *eagA* was further elucidated by the restoration of autoaggregation in the absence and presence of zinc, through the overexpression of *eagA* (p*eagA*OE vector) (Figure 10).

## Discussion

Our discovery of c-di-GMP-mediated regulation of zinc uptake in *E. amylovora* was dependent on our initial finding that *eagA* suppressed the autoaggregation phenotype in the Ea1189Δ*pdeABC* mutant. Our results suggest that EagA is a critical contributor to autoaggregation in *E. amylovora* that is observed when intracellular levels of c-di-GMP are high in the Ea1189Δ*pdeABC* mutant. This manifestation of autoaggregation is also specifically dependent on native *E. amylovora* c-di-GMP reserves, and not merely an increase in heterologously expressed c-di-GMP, indicating signaling specificity. The autoaggregation phenotype is also partially dependent upon the EPSs amylovoran and cellulose (Kharadi and Sundin, 2019). We demonstrated that transcript levels of *eagA* were significantly higher in Ea1189Δ*pdeABC* compared to the WT Ea1189, correlating with our observation of the involvement of EagA in autoaggregation. However, elevating *eagA* transcript levels in WT Ea1189 independently of the intracellular c-di-GMP levels, did not result in autoaggregation, suggesting that the regulation underlying autoaggregation is impacted by both the transcript levels of *eagA* as well as intracellular c-di-GMP levels. Concurrently, the complementation of autoaggregation through the use of expression vectors necessitated the use of an inducible overexpression vector (p*eagA*OE) to achieve high *eagA* transcript levels in addition to the existing high levels of c-di-GMP in Ea1189Δ*pdeABC* to achieve a phenotypic recovery of autoaggregation that was abrogated upon the deletion of *eagA*.

We also observed that EagA positively affected amylovoran production. Although *amsG* transcript levels were 3-fold higher in Ea1189Δ*pdeABC*Δ*eagA* compared to WT Ea1189, the deletion of *eagA* in the Ea1189Δ*pdeABC* background severely impaired amylovoran production as quantified in the supernatant. The reduction in amylovoran production was also observed in Ea1189Δ*eagA* compared to WT Ea1189. This suggests that the impact of the *eagA* deletion on amylovoran production may result from an inability of mutants to secrete amylovoran precursors, where they are polymerized into amylovoran through the action of periplasmic enzymes (Langlotz et al., 2011). Cell wall structural modifications caused as a result of peptidoglycan hydrolase activity have been shown to affect cell membrane dependent substrate transport in other systems (Nambu et al., 1999; Weber et al., 2016).

We also found that Ea1189Δ*eagA* was significantly reduced in virulence in apple shoots compared to WT Ea1189. Evidence exists linking the effect of cell wall remodeling due to peptidoglycan hydrolysis activity with the release of virulence factors at specific stages during infection of gram-positive bacterial pathogens (Bublitz et al., 2009; Rico-Lastres et al., 2015). In gram negative bacterial pathogens, lytic transglycosylases (Ltrs) play a similar role by cleaving glycosidic bonds within the bacterial peptidoglycan that allow for the insertion of cell membrane spanning structures such as flagella, type III secretion systems, and type IV pili (Zahrl et al., 2005). The role of Ltrs in pathogen virulence has been shown in a number of systems. For example, three genes from the plant pathogen *Pseudomonas syringae* pv. tomato that encoded proteins with predicted Ltr domains were shown to be coregulated with the type III secretion regulon, and mutants were reduced in type III effector translocation and virulence (Oh et al., 2007). Similarly, a putative Ltr designated HpaH was shown to be involved in the secretion of a set of type III effectors in *Xanthomonas campestris* (Buttner et al., 2007). We have also identified a putative Ltr-encoding gene, a homolog of *mltE* from *Yersinia pestis*, that was induced during infection of immature pear tissue by *E. amylovora* (Zhao et al., 2005). Regarding Ea1189Δ*eagA*, the virulence defect may be due to the reduction in production of amylovoran, which is a pathogenicity factor (Geider, 2000), but EagA may also be important for type III secretion.

Biofilm formation was positively impacted by the presence of *eagA* only under high intracellular levels of c-di-GMP found in Ea1189Δ*pdeABC.* Enzymes belonging to the endopeptidase class of peptidoglycan hydrolases have been shown to be necessary for the formation of normal biofilms due to their effect on cell separation during cell division and the maintenance of cell morphology through the modulation of peptidoglycan dynamics in *E. coli* (Priyadarshini et al., 2006; Ghosh et al., 2008). Potentially indicative of the sensitivity of biofilm formation to the level of enzymatic activity rendered by EagA, the overexpression of *eagA* was necessary to fully restore/surpass the basal level of biofilm formation recorded in Ea1189Δ*pdeABC.*

In contrast to biofilm formation, flagellar motility was found to be negatively regulated by *eagA*, independent of the relative intracellular concentration of c-di-GMP in the background strains. Motility, unlike autoaggregation and biofilm formation, required only a modest increase in *eagA* transcripts provided the p*eagA* vector for a complete phenotypic restoration in the mutants, suggesting a strong regulatory effect. A direct link between endopeptidase activity on the peptidoglycan layer and its impact on flagellar motility has not been established. However, in *S. enterica*, FlgJ (essential flagellar protein) functions as a β*-N-acetylglucosaminidase*, with peptidoglycan hydrolase properties. FlgJ was found to be necessary for proper flagellar assembly (Herlihey et al., 2014). Further research into the enzymatic impact of EagA on the peptidoglycan layer specifically impacting flagellar activity will provide some clarity about the functional dynamics of this preliminary observation.

In this study, we determined that the transcriptional activity of the *znuABC* zinc uptake system and the *eagA* peptidoglycan hydrolase is regulated by Zur and native *E. amylovora* generated c-di-GMP in response to the external presence or absence of Zn^2+^. Zur is a zinc-dependent repressor of the *znuAeagA* operon, and Zur is known to function to prevent zinc toxicity by hindering the excessive uptake of zinc by the cell (Patzer and Hantke, 2000; Gilston et al., 2014). Unexpectedly, when Zn^2+^ was amended in the medium, transcription of *znuAeagA* was significantly elevated under high c-di-GMP levels (in Ea1189Δ*pdeABC*), despite the availability of native Zur in this strain that would normally repress this transcriptional activity. Temporally, this transcriptional elevation was recorded very early after the introduction of zinc in the medium (15 min), with a subsequent increase in the magnitude of the response due to a sustained presence of zinc (2 h). This result indicates that c-di-GMP positively regulates transcription of the *znuAeagA* operon in *E. amylovora*, potentially through an associated transcriptional regulator. This underlying transcriptional regulatory effect on *znuAeagA* mediated by c-di-GMP, although also present in WT, is increased in Ea1189Δ*pdeABC* due to the lack of any regulatory feedback from Pde-mediated hydrolysis of intracellular c-di-GMP. Phenotypic corroboration of the effect of zinc availability on *eagA* transcription was observed in the form of an abrogation of the autoaggregation response in Ea1189Δ*pdeABC* due to the chelation of zinc. The presence of environmental Zn^2+^ is a prerequisite for the c-di-GMP mediated positive regulation of the *znuAeagA* operon. While *znuCB* operon expression followed a similar transcriptional pattern as *znuAeagA*, the only exception was the reduction of transcript levels in Ea1189Δ*pdeABC*Δ*zur* upon complementation with *zur* under high Zn^2+^ levels. *znuAeagA* transcript levels were consistently high and comparatively higher than *znuCB* (at 2 h), despite complementation with *zur* in Ea1189Δ*pdeABC*Δ*zur*, when Zn^2+^ was present in the environment. This suggests a transcriptional strand bias in the regulation mediated through c-di-GMP, leading to comparatively higher expression levels of *zurAeagA* vs. *znuCB*.

Zur-mediated transcriptional regulation of the *znuABC* gene cluster, and the associated zinc-dependent metallopeptidase encoding gene (*eagA* or *mepM*) has been studied in different bacterial models. This includes the transcriptional regulation of *mepM* in *E. coli*, which is located in an operon with *znuA*, as well as that of *shyB* in *V. cholerae*, which is transcribed as a separate gene (Graham et al., 2009; Murphy et al., 2019). However, c-di-GMP mediated transcriptional control of the *znuABC*/*eagA* gene cluster has not been reported previously. Although there are no other reports linking c-di-GMP and the regulation of zinc uptake, DgcZ, a c-di-GMP synthesizing DGC in *Escherichia coli*, has been shown to bind and to be allosterically regulated by zinc (Zahringer et al., 2013). DgcZ activity regulates the production of poly-β-1,6-N-acetyl-glucosamine (poly-GlcNAc)-dependent biofilm formation in uropathogenic *Escherichia coli*, and this response is specific to zinc compared to other divalent metal ions (Yeo et al., 2018). DgcZ contains a chemoreceptor zinc binding (CZB) domain that tightly binds Zn^2+^ ions (Ammendola et al., 2007). DGC activity, and thus poly-GlcNAc-dependent biofilm formation, is inhibited in the zinc-bound state. In a growth culture experiment, depletion of zinc levels from 1 mM to 2 μM stimulated DgcZ activity and c-di-GMP production, as monitored using a fluorescent biosensor (Yeo et al., 2018).

**FIGURE 11:**
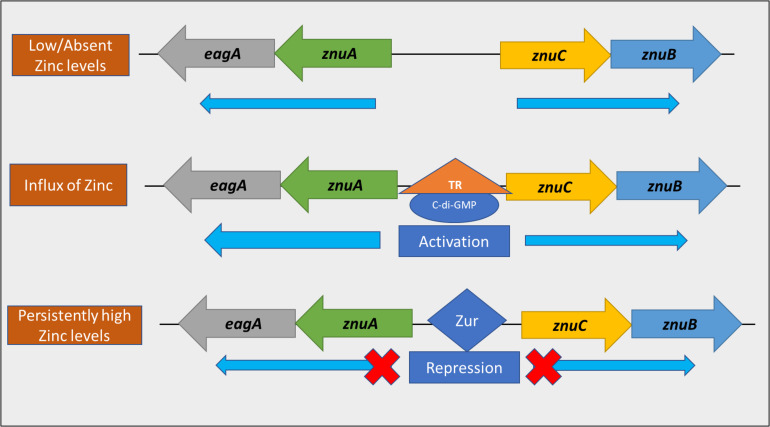
A model representing the dual transcriptional regulation of the *znuAeagA/znuBC* gene cluster. When the level of zinc in the environment is low, the Zur repressor is not bound to the bi-directional promoter within the gene cluster. Thus, transcription occurs at a basal level, bidirectionally. Upon the influx of zinc into the environment, transcriptional activation of the gene cluster occurs in a c-di-GMP dependent manner, potentially through an unknown transcriptional regulator (TR). This interaction strongly promotes transcription in the direction of *znuA*. As intracellular zinc levels increase, Zur binds to the promoter to prevent transcription, thus avoiding zinc toxicity.

Our model (Figure 11) suggests the presence of three regulatory stages in *E. amylovora* relevant to zinc uptake from the environment. When zinc is low/absent in the environment, Zur is not bound to the bi-directional promoter located between *znuAeagA and znuCB*, and transcription in both directions would proceed at the basal rate, driven by σ^70^ promoter sequences. For example, in *Salmonella enterica* serovar Typhimurium, production of the ZnuA protein was detected after growth in minimal medium containing 0.1 μM or less added ZnSO_4_, and was not detected when the medium contained 0.5 μM or more added ZnSO_4_ (Ammendola et al., 2007). Upon the influx of zinc in the environment, transcriptional activation of *znuAeagA and znuCB* in *E. amylovora* occurs bidirectionally in a c-di-GMP dependent manner potentially through an unknown transcriptional regulator, with a preferential strand bias in the *znuAeagA* direction. This, we hypothesize, is to promote zinc acquisition in this early stage. This transcriptional activation gets alleviated primarily due to a reduction in the levels of c-di-GMP available as a regulatory substrate, as a result of hydrolysis by one or more Pdes in *E. amylovora*. Our experimental setup involving the use of Ea1189Δ*pdeABC* both skews the level of transcriptional activation at this early stage, and, delays the suppression of this response, due to the absence of any functional Pde enzymes. The secondary stage of regulation involves transcriptional repression as a result of the zinc-bound form of Zur binding to the promoter, with the objective of potentially avoiding zinc toxicity. The currently understood regulatory model does not include this new intermediate stage of regulation mediated by c-di-GMP. Additionally, our data suggests that in the absence of Zur, the transcriptional effect mediated by c-di-GMP is enhanced further, in addition to the existent elevated positive regulation occurring due to the high intracellular levels of c-di-GMP within Ea1189Δ*pdeABC*. Determining the region within the *znuA* promoter that is involved in c-di-GMP mediated transcriptional activation, and, knowing its location relative to the Zur binding box will be critical to fully understand how the transition from transcriptional activation to repression occurs.

Zur contains specific zinc binding sites that regulate either protein stability or DNA binding affinity (Shin et al., 2011). In addition, Zur-mediated transcriptional regulation is governed by the native binding affinity of the promoter region to Zur, as well as the level of zinc that the cell is treated with (Shin et al., 2011; Gilston et al., 2014). Additionally, Zur requires zinc to be present intracellularly for binding (Patzer and Hantke, 2000), making this process dependent on zinc import through the ZnuABC system, prior to initiation of the feedback regulation from Zur. Thus, we speculate that the c-di-GMP mediated regulation at the *znuA* promoter primarily operates during the regulatory gap after Zur is released from the promoter due to prolonged exposure to depleted zinc levels in the environment and before zinc levels increase to a point where zinc-bound Zur again binds the promoter. Our data suggest that, following the influx of zinc into cells that were previously zinc-depleted, c-di-GMP-mediated transcriptional induction of *znuA/eagA* and *znuCB* occurs. The binding and transcriptional regulation of the promoter by a putative c-di-GMP dependent transcriptional regulator might be highly sensitively to intracellular levels of c-di-GMP, which would be regulated by the action of a Pde (Romling et al., 2013). This would explain why *znuA* transcription proceeds strongly in Ea1189Δ*pdeABC* despite the presence of Zur, 2 h after exposure to 200 nm ZnSO_4_. Thus, further studies will be necessary to fully understand the shifting transcriptional dynamics during the process of zinc sensing and zinc uptake involving the dual regulation of the *znuA* promoter by Zur and c-di-GMP.

Research on c-di-GMP signaling continues to indicate the importance of this second messenger molecule in controlling the regulation of ecologically important traits in a large diversity of bacteria (Romling et al., 2013). While the most common c-di-GMP regulated traits include biofilm formation, motility, and other determinants involved in pathogen virulence (Valentini and Filloux, 2019), there is only limited information available indicating c-di-GMP regulation of metal uptake. The most prominent example currently stems from observations in *Pseudomonas aeruginosa* that reveal a link between the Gac/Rsm system, c-di-GMP, and the synthesis of pyoverdine, a siderophore involved in iron uptake (Frangipani et al., 2014) with at least four Dgcs involved in supplying the c-di-GMP that induces pyoverdine synthesis (Chen et al., 2015). Since the Gac/Rsm regulon is the major controlling regulator of the switch from planktonic to biofilm lifestyle, c-di-GMP signaling couples increased iron uptake to biofilm development. This is corroborated by knowledge that iron uptake is required for biofilm formation in *P. aeruginosa* under iron-limited conditions, and that *P. aeruginosa* biofilm cells are more susceptible to iron limitation than planktonic cells (Banin et al., 2005; Patriquin et al., 2008). Owing to the critical nature of the metals like iron and zinc in regulating multiple bacterial cellular processes, and their varied and fluctuating zonal distribution occurring within the plant host (Marschner, 1993; Lorenz et al., 1994; Reid et al., 1996), we hypothesize that the regulatory effect on zinc uptake mediated by c-di-GMP is potentially part of a larger pathogenic adaptation by *E. amylovora* within the host. Zinc could serve as a sensory cue within the host that could trigger a wide range of regulatory responses within *E. amylovora* that contribute to metabolic and virulence functions.

Overall, we found that EagA contributes to autoaggregation in a c-di-GMP dependent manner. EagA levels also contribute positively to amylovoran production and virulence in apple shoots. Also, *eagA* is located in an operon with *znu*A; thus, *eagA* expression is associated with that of the *znuABC* zinc uptake gene cluster. The *znuAeagA*/*znuBC* gene cluster is regulated through the action of both Zur-mediated transcriptional repression and c-di-GMP-mediated transcriptional activation. The c-di-GMP mediated regulation occurs upon the influx of zinc in the environment to potentially rapidly promote zinc uptake. Further study is needed to elucidate the role of zinc in the biology and pathogenesis of *E. amylovora*.

## Data Availability Statement

All datasets presented are included in the study.

## Author Contributions

RK and GS planned and designed the components of the study and edited and finalized the manuscript. RK conducted experiments, conducted related data analysis, and wrote the manuscript. Both authors contributed to the article and approved the submitted version.

## Conflict of Interest

The authors declare that the research was conducted in the absence of any commercial or financial relationships that could be construed as a potential conflict of interest.

## Supplementary Material

Supplementary Figure 1 Intracellular levels of c-di-GMP (nM) quantified via. UPLC-MS-MS in WT Ea1189, Ea1189Δ*pdeABC*, and *eagA*/*zur* mutants constructed in each of the two background strains along with complemented strains. The data includes at three biological replicates with error bars representing standard error of the means. Differential letters above the bars indicate statistically significant differences [*P* < *0.05* by Tukey’s honestly significant difference (HSD) test] within each experimental condition subgroup.

Supplementary Figure 2 Nucleotide sequence alignment of the **(A)** intergenic region between *znuA* and *znuC* in *E. coli* K12 W3110 and *E. amylovora* ATCC-49946, **(B)** M23 peptidase and **(C)** LysM domain residues in EagA, MepM, and Shy B using MEGA version 7.0 (Kumar et al., 2016) via clustal omega (Sievers et al., 2011). Asterisks above the alignment mark the conserved residues and the dashes along the aligned sequences represent the relative gaps after alignment. Zur-interacting nucleotides in *Escherichia coli*, as demonstrated by Gilston et al. (2014), are boxed.

Supplementary Figure 3 *eagA*, *znuA*, *znuC*, and *znuB* expression levels in WT *E. amylovora* Ea1189 and Ea1189 expressing pVC_DGCOE. Strains were grown in LB for 18 h with IPTG as appropriate. Data presents relative fold change in each of the target genes for Ea1189 compared to Ea1189/pVC_DGCOE. Data includes three biological replicates, error bars representing the standard error of the means. Asterisk above the bars indicate statistically significant differences in expression [*P* < *0.05* by student’s *t*-test] within each gene target subgroup.

Supplementary Table 1 Oligonucleotides and primers used in this study.
