# Supplementary figures and images for: Cyclic-di-GMP Regulates Autoaggregation Through the Putative Peptidoglycan Hydrolase, EagA, and Regulates Transcription of the znuABC Zinc Uptake Gene Cluster in Erwinia amylovora
Source: Front Microbiol. 2020 Nov 17;11:605265. doi: 10.3389/fmicb.2020.605265 (PMC7705223; doi:10.3389/fmicb.2020.605265)

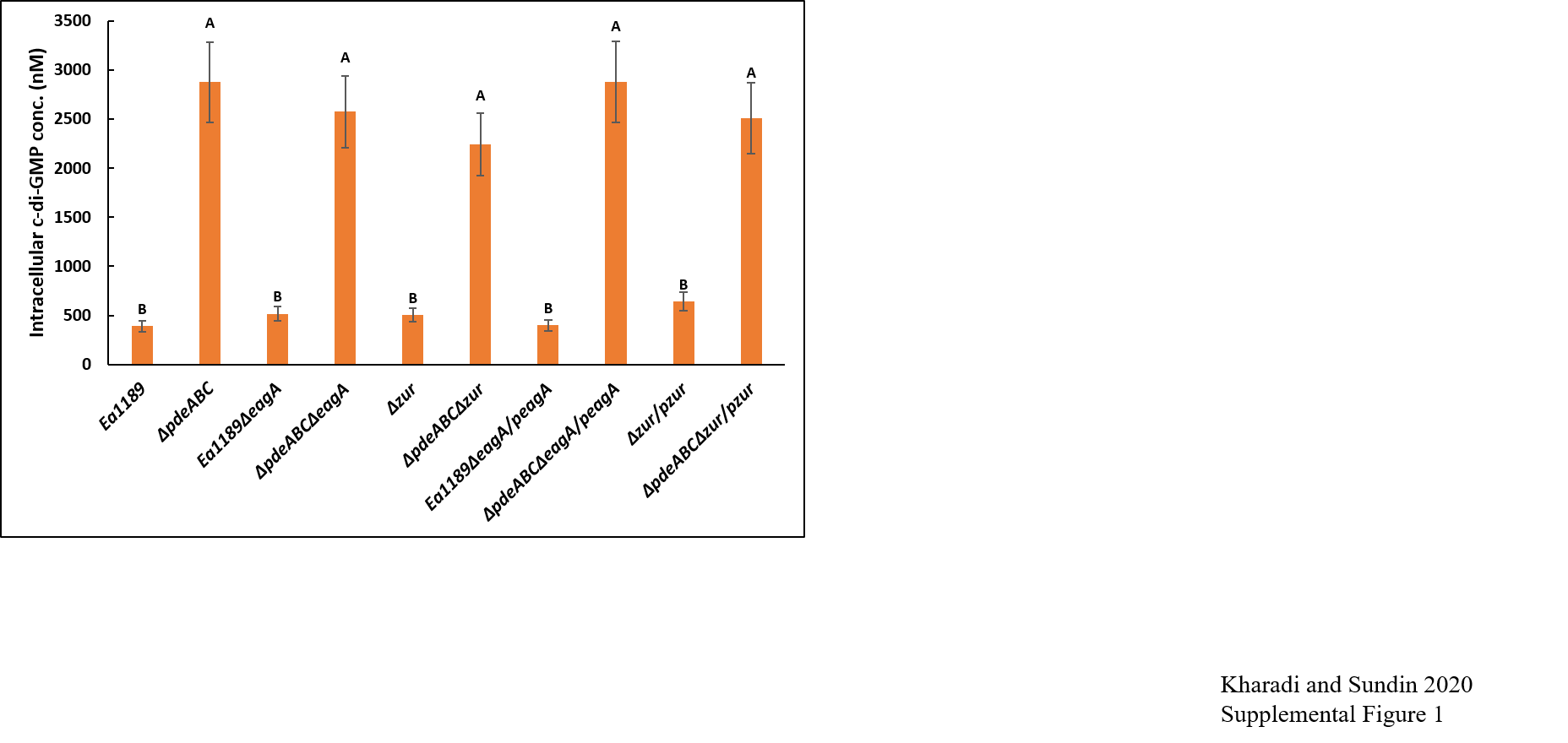

Supplement: Supplementary Figure 1 — Intracellular levels of c-di-GMP (nM) quantified via. UPLC-MS-MS in WT Ea1189, Ea1189ΔpdeABC, and eagA/zur mutants constructed in each of the two background strains along with complemented strains. The data includes at three biological replicates with error bars representing standard error of the means. Differential letters above the bars indicate statistically significant differences [P < 0.05 by Tukey’s honestly significant difference (HSD) test] within each experimental condition subgroup. [file Image_1.TIF]

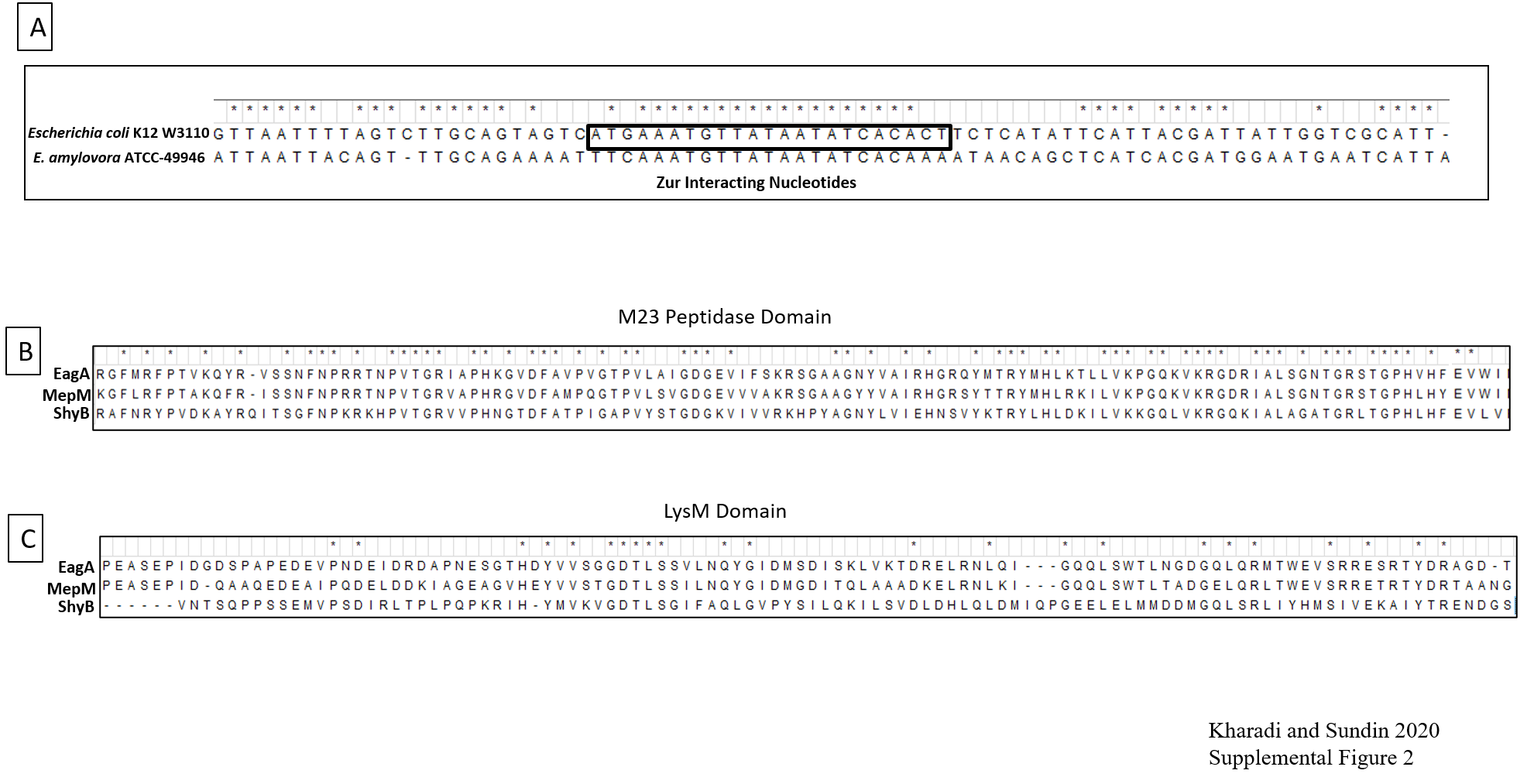

Supplement: Supplementary Figure 2 — Nucleotide sequence alignment of the (A) intergenic region between znuA and znuC in E. coli K12 W3110 and E. amylovora ATCC-49946, (B) M23 peptidase and (C) LysM domain residues in EagA, MepM, and Shy B using MEGA version 7.0 (Kumar et al., 2016) via clustal omega (Sievers et al., 2011). Asterisks above the alignment mark the conserved residues and the dashes along the aligned sequences represent the relative gaps after alignment. Zur-interacting nucleotides in Escherichia coli, as demonstrated by Gilston et al. (2014), are boxed. [file Image_2.TIF]

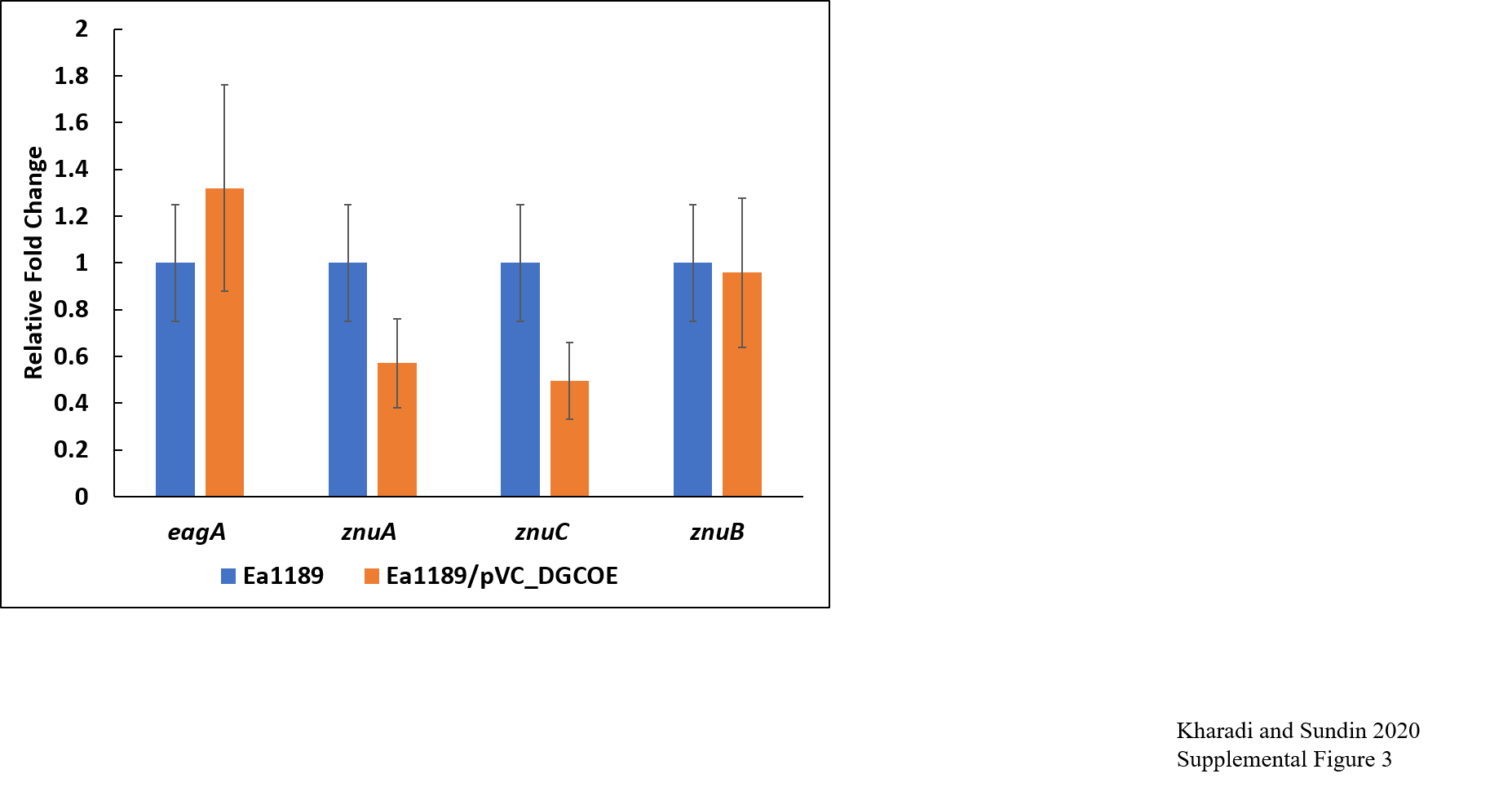

Supplement: Supplementary Figure 3 — eagA, znuA, znuC, and znuB expression levels in WT E. amylovora Ea1189 and Ea1189 expressing pVC_DGCOE. Strains were grown in LB for 18 h with IPTG as appropriate. Data presents relative fold change in each of the target genes for Ea1189 compared to Ea1189/pVC_DGCOE. Data includes three biological replicates, error bars representing the standard error of the means. Asterisk above the bars indicate statistically significant differences in expression [P < 0.05 by student’s t-test] within each gene target subgroup. [file Image_3.TIF]
