# Supplementary material for: Cyclic-di-GMP Regulates Autoaggregation Through the Putative Peptidoglycan Hydrolase, EagA, and Regulates Transcription of the znuABC Zinc Uptake Gene Cluster in Erwinia amylovora
Source: Front Microbiol. 2020 Nov 17;11:605265. doi: 10.3389/fmicb.2020.605265 (PMC7705223; doi:10.3389/fmicb.2020.605265)
Supplement: Supplementary Table 1 — Oligonucleotides and primers used in this study. [file Table_1.pdf]

Supplemental Table 1. Oligonucleotides and primers used in this study

Primer	Sequence	Use	Reference
eagA mut Fw	GTGCAGCAAAAACTCAACGCTATCAGCGTAGCGTTTAATGGTTTATCCCGCCCCCATGTGTAGGCTGGAGCTGCTTC	*eagA* chromosomal deletion primer	This study
eagA mut Rv	CTACCTGATGCGCAGCTGGGGGATGACTTCACGCACCTGTGCCAGATAATCACGGCGCTCATATGAATATCCTCCTTA	*eagA* chromosomal deletion primer	This study
zur mut Fw	ATGACCAGCGTTGAAAAAATTCTGACGCAGGCAGAAATTTTCTGGTGTAGGCTGGAGCTGCTTC	*zur* chromosomal deletion primer	This study
zur mut Rv	TTATCGCCCACGTTTCTTACTCTGTACGCTGTGGTCGTGATGCATATGAATATCCTCCTTA	*zur* chromosomal deletion primer	This study
eagA pBBR1 Fw	AAAACTCGAGGTACAGATATCTCTCCAGGCAAAGACAGCTACGTGC	*eagA* cloning primer for pBBR1MCS5	This study
eagA pBBR1 Rv	AAAATCTAGAGACGTGGGATTTTGCAATGGTAACGGTCAAAGCGCCCATTCA	*eagA* cloning primer for pBBR1MCS5	This study
zur pBBR1 Fw	AAAACTCGAGTTCAGTGAAGATAAGGGGGCTGATTGG	*zur* cloning primer for pBBR1MCS5	This study
zur pBBR1 Rv	AAAATCTAGAGCCTTGACAGGCACAAGGACAGTGCCAC	*zur* cloning primer for pBBR1MCS5	This study
eagA pEVS Fw	AAAAGAATTCGTGCAGCAAAAACTCAACGCTATCAGCGTAGCGTTTAATGGTTTATCCCG	*eagA* cloning primer for pEVS143	This study
eagA pEVS Rv	AAAACTCGAGCTACCTGATGCGCAGCTGGGGGATGACTTCACGCACCTGTG	*eagA* cloning primer for pEVS143	This study
DGEN-1	GGCCACGCGTCGACTAGTCAGNNNNNNNNNNACGCC	Arbitrary PCR/Tn insertion site ID	(Miller-Williams et al., 2006)
DGEN-2	GGCCACGCGTCGACTAGTCAG	Arbitrary PCR/Tn insertion site ID	(Miller-Williams et al., 2006)
IS50A	CACGATGAAGAGCAGAAG	Arbitrary PCR/Tn insertion site ID	(Miller-Williams et al., 2006)
IS50B	TAGGAGGTCACATGGAAGTCAGAT	Arbitrary PCR/Tn insertion site ID	(Oresnik et al., 1998)
TetR ext	ATGGTAAAATAACTCTATCAATG	Arbitrary PCR/Tn insertion site ID	This study
TetR int	AGTTTTTAAGCACATCATCATC	Arbitrary PCR/Tn insertion site ID	This study
q-RT- PCR primers			
amsG fw	CTGCCACAATCCGGTCATGC	q-RT-PCR	(Kharadi et al., 2019)
amsG rv	TGCCGCAACTATGGAACGTG	q-RT-PCR	(Kharadi et al., 2019)
znuA fw	GGCTCAGGCTGCCATTGTTG	q-RT-PCR	This study
znuA rv	GGTTACGCCTTCGGCAATCG	q-RT-PCR	This study
eagA fw	GTGATGGCCAGCTACAGCGT	q-RT-PCR	This study
eagA rv	GGTCGTAGGTGCGGCTTTCA	q-RT-PCR	This study
znuB fw	GGAATGGTGGTGGTCAGCCT	q-RT-PCR	This study
znuB rv	GCCAGCAGATCGCCAAACAG	q-RT-PCR	This study
znuC fw	TACAGCCGGGCCGAATACTG	q-RT-PCR	This study
znuC rv	GGGTGCGATAAGTCCCAGCA	q-RT-PCR	This study
